# Mucoadhesive effect of *Curcuma longa* extract and curcumin decreases the ranitidine effect, but not bismuth subsalicylate on ethanol-induced ulcer model

**DOI:** 10.1038/s41598-019-53089-2

**Published:** 2019-11-12

**Authors:** Alejandra Orona-Ortiz, Luis Medina-Torres, Josué A. Velázquez-Moyado, Elizabeth A. Pineda-Peña, José Luis Balderas-López, María Josefa Bernad-Bernad, José Carlos Tavares Carvalho, Andrés Navarrete

**Affiliations:** 1 Facultad de Química, Departamento de Farmacia, Universidad Nacional Autónoma de México. Ciudad Universitaria Coyoacán, 04510 Ciudad de Mexico, Mexico; 2 Laboratorio de Pesquisa em Farmacos, Curso de Farmacia, Departamento de Ciências Biólogicas e da Saúde, Universidade Federal do Amapá, Macapá, AP Brazil

**Keywords:** Peptic ulcers, Natural products

## Abstract

The study of pharmacological interactions between herbal remedies and conventional drugs is important because consuming traditional herbal remedies as supplements or alternative medicine is fairly common and their concomitant administration with prescribed drugs could either have a favorable or unfavorable effect. Therefore, this work aims to determine the pharmacological interactions of a turmeric acetone extract (TAE) and its main metabolite (curcumin) with common anti-ulcer drugs (ranitidine and bismuth subsalicylate), using an ethanol-induced ulcer model in Wistar rats. The analysis of the interactions was carried out via the *Combination Index-Isobologram Equation* method. The combination index (*CI*) calculated at 0.5 of the affected fraction (*fa*) indicated that the TAE or curcumin in combination with ranitidine had a subadditive interaction. The results suggest that this antagonistic mechanism is associated to the mucoadhesion of curcumin and the TAE, determined by rheological measurements. Contrastingly, both the TAE and curcumin combined with bismuth subsalicylate had an additive relationship, which means that there is no pharmacological interaction. This agrees with the normalized isobolograms obtained for each combination. The results of this study suggest that mucoadhesion of curcumin and the TAE could interfere in the effectiveness of ranitidine, and even other drugs.

## Introduction

For centuries, medicinal plants were the primary source of health care. Herbal remedies are still used by ~70% of the population, according to the World Health Organization (WHO)^1^. Some studies have reported that despite the lack of information about the efficacy and safety regarding alternative and traditional herbal medicine, their use is increasing. Therefore, it is essential to investigate possible interactions between herbal treatments and conventional drugs. When simultaneously consuming herbal and drug treatments, there may be negative interactions that diminish the effectiveness of the treatment, or could even present adverse symptomatology^1–3^. However, some interactions could be favorable to the patient, enhancing the therapeutic activity^4^.

**Figure 1:**
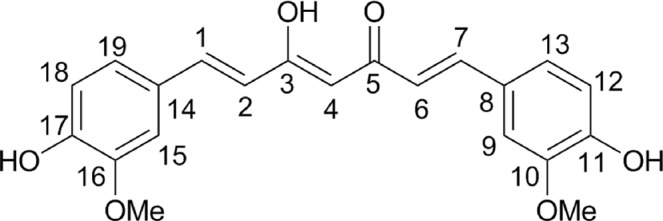
Chemical structure of curcumin.

Turmeric root (*Curcuma longa* L. [Zingiberaceae]) is used in Ayurveda and Chinese traditional medicine as a remedy for peptic ulcer^5^. Several studies show the gastroprotective activity of *Curcuma* extracts in different ulcer models in rats. These extracts also accelerate the gastric mucosal healing, decrease the acid release and increase the expression of cyclooxygenase-2 (COX-2) and superoxide dismutase (SOD)^6–8^. Curcumin (Fig. 1), one of the major components of *C. longa*^9,10^, improves ulceration healing by the restitution of collagen fibers and angiogenesis stimulation^11^. It has been reported that the ethyl acetate extract of *Curcuma longa* prevents the gastric mucosal damage in pylori-ligated rats, decreasing the acid release by antagonizing histamine-2 receptors; however, curcumin does not show this antagonistic effect^6^. Nevertheless, the antioxidant effect of curcumin could help to prevent ethanol-induced gastric damage due to its scavenging activity on reactive oxygen species (ROS)^12,13^.

Peptic ulceration is defined as a gastric mucous membrane injury in the stomach or duodenum, with the sloughing of inflamed dead tissue^14^. In Mexico, around 1’500,000 cases of gastric ulcer, gastritis and duodenitis are annually reported^15^. Despite that Proton Pump Inhibitors (PPIs) are the most common worldwide treatment for peptic ulcer, it is reported that omeprazole increases duodenal ethanol injury in rats^16^. Under our experimental conditions, using an ethanol-induced gastric damage model, omeprazole, did not show a dose-dependent effect, so construction of the dose-response curve was not possible. Therefore, in this study, we decided to evaluate another over-the-counter (OTC) drugs used in ulcer treatment. Ranitidine is a histamine-2 receptor antagonist, which decreases acid secretion^17^, and bismuth subsalicylate protects the gastric mucosa from damage by covering and protecting the stomach from luminal acid and increases the mucus secretion^18^; it has also been reported that it protects against stress, an inadequate diet and alcohol-induced injuries^19^.

Additionally, the turmeric market has increased around the world^5^; therefore, the concomitant use of curcuma with conventional antiulcer drugs is likely among patients with gastric ailments. It is of great importance to study pharmacological interactions because it is not usually known how the organism will respond to a multi-drug treatment. The effect of a given combination may be simply the addition of the individual effects (additivity), or there could be an increase or attenuation of the activity. The enhanced effect is called *superadditive* or *synergistic* (favorable interactions), and the attenuated one *subadditive* (unfavorable interactions)^20^. Favorable interactions may help to diminish the drug doses and their side effects. When finding an unfavorable interaction, drug combinations should be adequately handled.

Continuing with our systematic study on the pharmacological interactions between natural products and conventional drugs^21–23^; in this study, we investigated the acetone extract of *C. longa* (TAE) and curcumin, its major active metabolite, in combination with ranitidine and bismuth subsalicylate, using an ethanol-induced gastric injury model in Wistar rats^24^. We used the *Combination Index-Isobologram Equation* analysis described by Chou and Talalay in 1976 to assess the effects of the combined treatments^20^.

## Results

**Figure 2:**
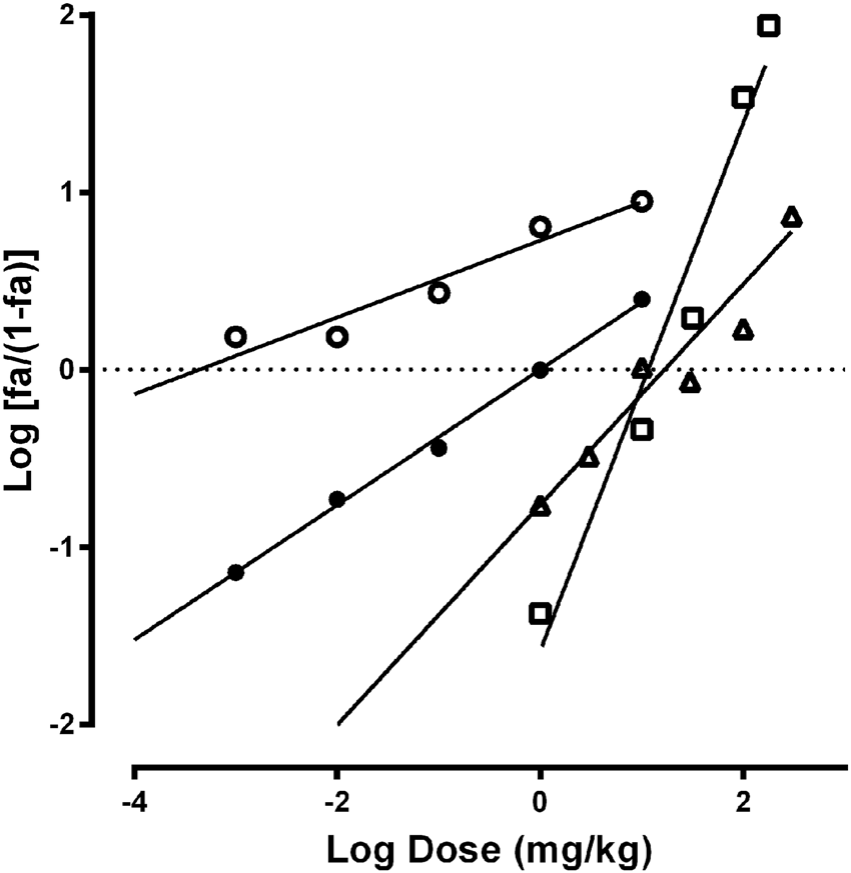
Median effect-plot of the single treatments: TAE (○) *Dm*: 0.004 mg/kg, curcumin (●) *Dm*: 0.99 mg/kg, ranitidine (**△**) *Dm*: 17.40 mg/kg and bismuth subsalicylate (□) *Dm*: 11.50 mg/kg.

**Figure 3:**
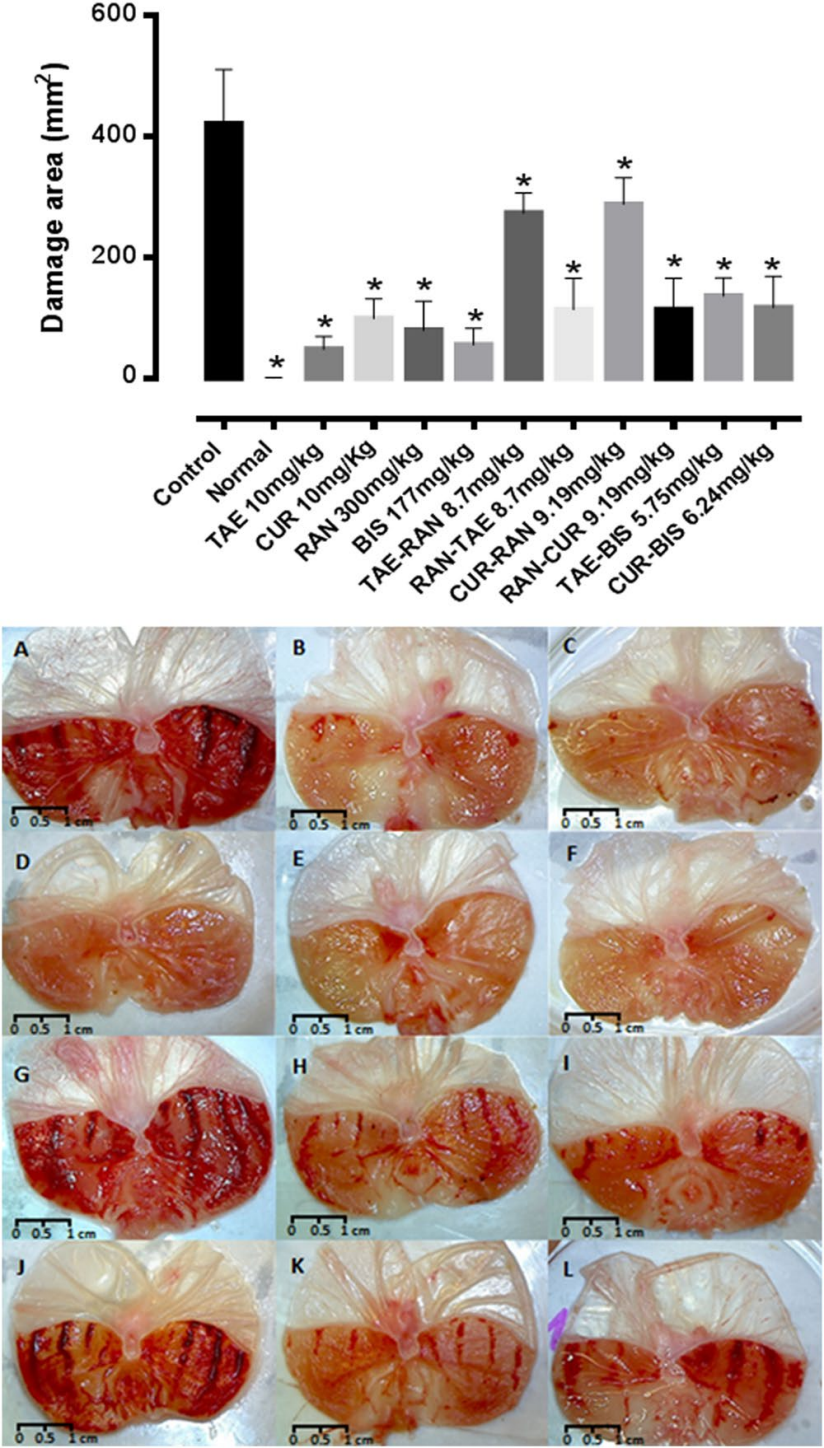
Damage areas (mm^2^) of the representative treatments. *The statistically significant difference (*p* < 0.05) between the treatments and the control was determined by a One-way ANOVA and a *post hoc* Dunnett test. Representative stomachs for each treatment: (**A**) control (absolute ethanol); (**B**) TAE (10 mg/kg), (**C**) curcumin **(**10 mg/kg); (**D**) normal stomach; (**E**) ranitidine (300 mg/kg); (**F**) bismuth subsalicylate (177 mg/kg); (**G**) TAE-ranitidine 1:1 and (**H**) ranitidine-TAE 1:1 (*Dmteo* 8.7 mg/kg); (**J**) curcumin-ranitidine 1:1 and (**K**) ranitidine-curcumin 1:1 (*Dmteo* 9.19 mg/kg); (**I**) TAE-bismuth subsalicylate 1:1 (*Dmteo* 5.75 mg/kg) and (**L**) curcumin-bismuth subsalicylate 1:1 (*Dmteo* 6.24 mg/kg*)*.

The curcumin and TAE were more potent inhibitors of the gastric damage (Table 1), with lower *Dm* values (TAE 0.004 mg/kg; curcumin 0.99 mg/kg) than those of ranitidine and bismuth subsalicylate (17.4 and 11.5 mg/kg). The calculated *Dm* were used to establish the dose-proportion in each combination, according to the experimental design (Table 2). The median-effect plots for each combination (Fig. 2) were used to calculate the *Dm* and *m* using Eq. 4 for both combinations (Table 1). Figure 3 shows representative pictures of stomachs for each treatment, in which the macroscopic gastric damage caused by absolute ethanol is observed. All treatments showed less damaged area (mm^2^) than the ulcerated control group also, it is important to notice that the combinations TAE-ranitidine and curcumin-ranitidine showed more damaged area than the other treatments.

**Table 1:** Median dose (*Dm*) and graph shape (*m*) obtained from the *median-effect plot*, for each drug and combination.

	Dm Exp (mg/kg)	Dm Theo (mg/kg)	m	CI_10_		CI_50_		CI_90_	
TAE	0.004		0.21						
Curcumin	0.99		0.38						
Ranitidine	17.40		0.62						
Bismuth subsalicylate	11.50		1.49						
TAE-Ranitidine	31.11	8.70	1.19	7196	Ant	3.57	Ant	0.32	Syn
Curcumin-Ranitidine	31.79	9.19	0.74	27.65	Ant	3.37	Ant	1.12	Add
TAE-Bismuth subsalicylate	6.35	5.75	0.98	1499	Ant	1.10	Add	1.17	Add
Curcumin-Bismuth subsalicylate	8.02	6.24	1.16	31.35	Ant	1.28	Add	0.99	Add

Combination index (*CI*) values for low (*CI*_10_), medium (*CI*_50_) and high (*CI*_90_) level of *fa* effect. *Dm Exp* and *Dm Theo* are the experimental and theoretical *Dm* values in the combinations. *Add* indicates additive effect (*CI* = 1), *Ant* indicates antagonism (*CI* > 1) and *Syn* indicates synergism (*CI* < 1). *CI*_10_, *CI*_50_, and *CI*_90_ are the *CI* calculated for the combinations at 0.1, 0.5 and 0.9 affected fractions (*fa*).

**Table 2:** Experimental design for the evaluation of 1:1 combinations.

			*Drug 1*				
		0	0.25 × (*Dm*)_1_	0.5 × (*Dm*)_1_	(*Dm*)_1_	2 × (*Dm*)_1_	4 × (*Dm*)_1_
	0	Control	(*fa*)_1_	(*fa*)_1_	(*fa*)_1_	(*fa*)_1_	(*fa*)_1_
		(*fa*)_0_					
*Drug 2*	0.25 × (*Dm*)_2_	(*fa*)_2_	(*fa*)_1,2_				
	0.5 × (*Dm*)_2_	(*fa*)_2_		(*fa*)_1,2_			
	(*Dm*)_2_	(*fa*)_2_			(*fa*)_1,2_		
	2 × (*Dm*)_2_	(*fa*)_2_				(*fa*)_1,2_	
	4 × (*Dm*)_2_	(*fa*)_2_					(*fa*)_1,2_

*Dm* = median dose; *fa* = affected fraction. Drug 1 = curcumin or TAE. Drug 2 = ranitidine or bismuth subsalicylate.

**Figure 4:**
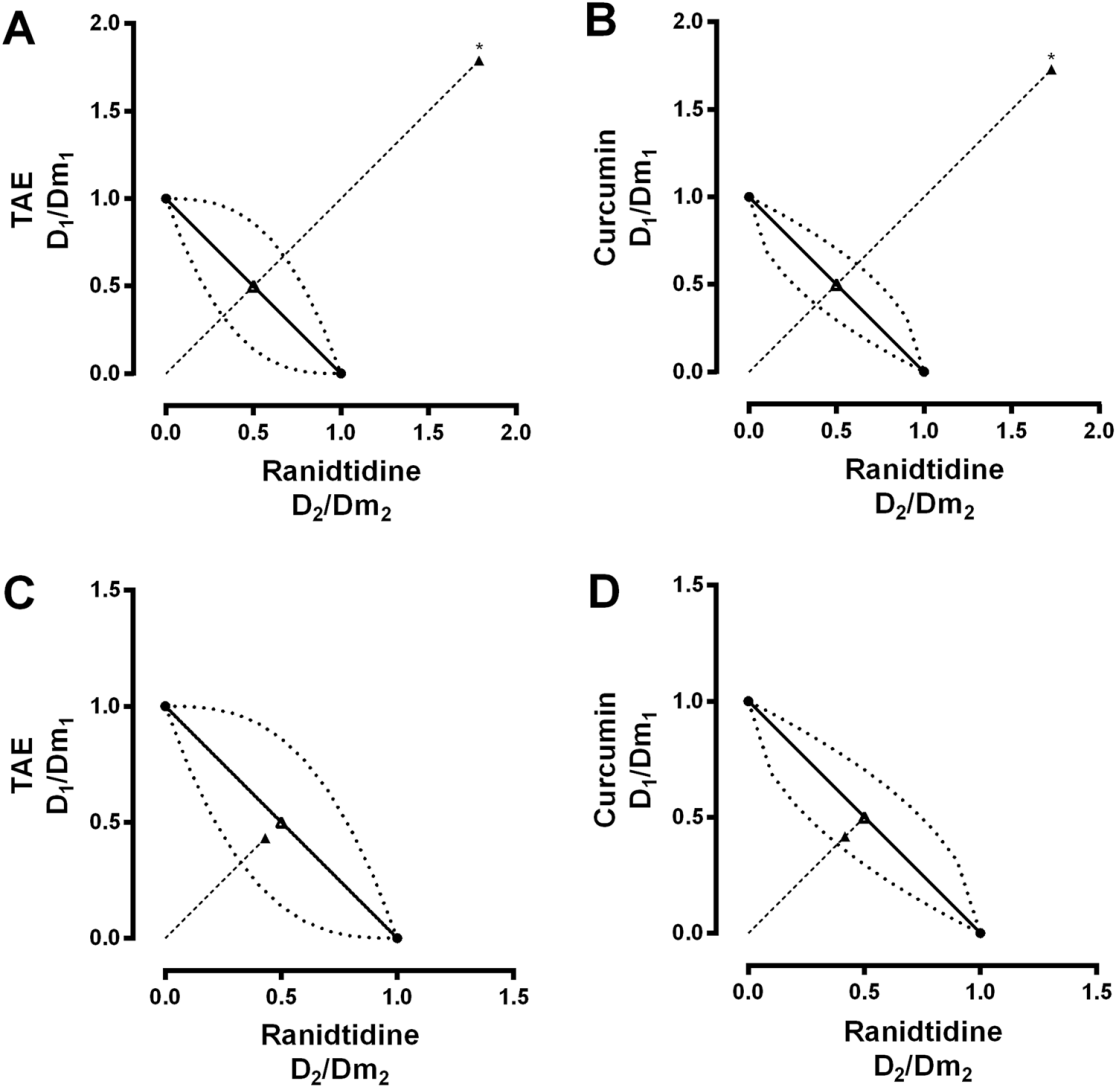
Normalized isobolograms of *Dm* at a 1:1 constant ratio of (**A**) TAE-ranitidine, (**B**) curcumin-ranitidine, (**C**) ranitidine-TAE (inverse administration) and (**D**) ranitidine-curcumin (inverse administration). The experimental *Dm* points calculated for the combinations (▲) were plotted and compared *vs* the theoretical *Dm* (**△**). *The statistically significant difference (*p* < 0.05) between the experimental and theoretical *Dm* was determined by the Mann-Whitney test. The concave curve and convex curve in the isobologram were calculated using Eqs 6 and 7.

**Figure 5:**
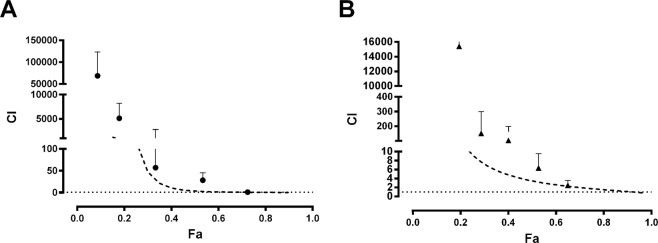
Calculated combination index (*CI*) for (**A**) TAE-ranitidine 1:1 (●) and (**B**) curcumin-ranitidine 1:1 (▲). Dashed line represents the theoretical *CI* behavior (–). *CI* shows synergism, additivity or antagonism at *CI* < 1, *CI* = 1 or *CI* > 1, respectively. Each point represents the mean ± SEM of at least six repetitions.

When TAE and curcumin were administrated before ranitidine, the combinations showed a subadditive interaction (Figs 3 and 4A,B). However, when the administration sequence was inverted, there was an additive effect (Fig. 4C,D). Figure 5 shows the combination index (*CI*) calculated for the combinations of TAE and curcumin with ranitidine. The *CI* for 0.5 *fa* indicates there is a subadditive interaction; which agrees with the isobolographic analysis (Fig. 4).

**Figure 6:**
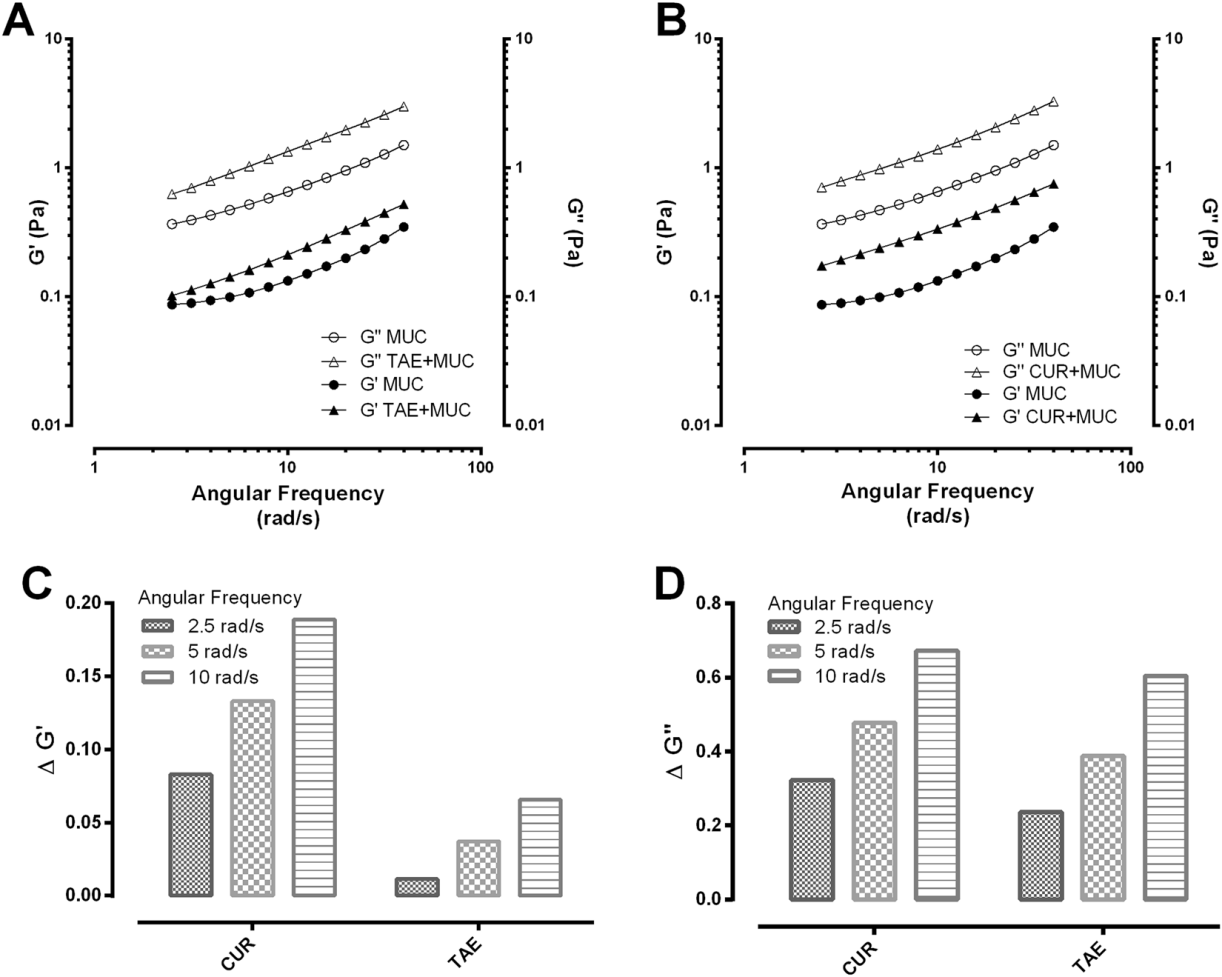
Frequency dependence graphics of the elastic (Gʹ) and viscous (Gʹʹ) modulus for the mixtures of mucin with TAE (**A**) or curcumin (**B**), compared to mucin alone. (**C**,**D**) show the synergy parameters (ΔGʹ and ΔGʹʹ) at different angular frequencies (2.5, 5 and 10 rad/s) for TAE and curcumin mixtures with mucin.

The observed subadditive effect may be attributed to the fact that TAE and curcumin coat the mucosal layer of the stomach, forming a protective barrier. This barrier could interfere with ranitidine absorption, thus reducing its gastroprotective effect. To further analyze the antagonist behavior of the TAE and curcumin against the ranitidine effect, we decided to indirectly evaluate the mucoadhesion. Figure 6 shows that mixtures of TAE-mucin and curcumin-mucin have rheological synergy (positive ΔGʹ and ΔGʹʹ). This indicates a strong interaction between the components of the mixture, which is related to mucoadhesion. The oscillatory flux-plots (Fig. 6A,B) show a “pseudo-solid” behavior under the testing conditions at all frequencies (ΔGʹʹ > ΔGʹ), suggesting a strong interaction between the continuous and dispersed phases. The higher synergy values were observed at high frequencies (Fig. 6C,D).

**Figure 7:**
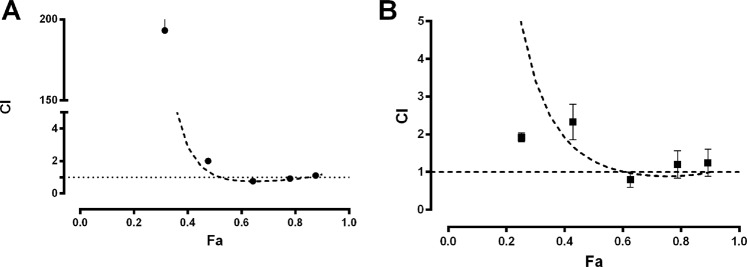
Calculated combination index (*CI*) for (**A**) TAE-bismuth subsalicylate 1:1 (●) and (**B**) curcumin-bismuth subsalicylate 1:1 (■). Dashed line represents the theoretical *CI* behavior (–). *CI* shows synergism, additivity or antagonism at *CI* < 1, *CI* = 1 or *CI* > 1, respectively. Each point represents the mean ± SEM of at least six repetitions.

**Figure 8:**
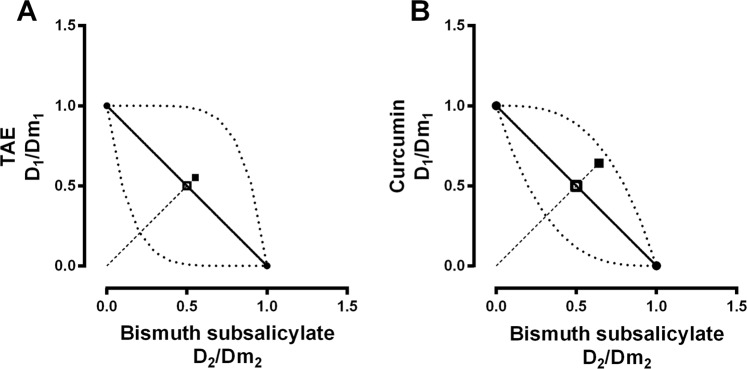
Normalized isobolograms of *Dm* at a 1:1 constant ratio of (**A**) TAE-bismuth subsalicylate and (**B**) curcumin-bismuth subsalicylate. The experimental *Dm* points calculated for both combinations (■) were plotted and compared *vs* the theoretical *Dm* (□). *The statistically significant difference (*p* < 0.05) between the experimental and theoretical *Dm* was determined by the Mann-Whitney test. The concave curve and convex curve in the isobologram were calculated using Eqs 6 and 7.

The combination of curcumin or TAE with bismuth subsalicylate does not show any pharmacological interaction. The *CI*-plots of these combined treatments (Fig. 7) correspond to an additive interaction for *fa* ≥ 0.5 and subadditive for *fa* < 0.5. The normalized isobolograms of the bismuth subsalicylate with TAE and curcumin combined treatments showed an additive effect in both cases (Fig. 8). The experimental *Dm* falls within the additive area of the isobolograms and there is no statistical difference between the experimental (*Dm Exp*) and theoretical *Dm* (*Dm Theo*) (Table 1).

## Discussion

The gastroprotective effect of turmeric and ranitidine proceeds via different mechanisms of action. For the *Curcuma* species, the molecular mechanism involves antioxidant and anti-inflammatory activities, regulating the most important inflammatory modulators: the inducible nitric oxide synthase (iNOS), tumor necrosis factor-alpha (TNF-α) and nuclear factor kappa-B (NF-κB)^10^. Ranitidine is an antagonist of the histamine-2 receptors that reduces the secretion of hydrochloric acid and pepsin^17^. Interestingly, Kim et al. (2005) reported that the *Curcuma longa* ethyl acetate extract decreases the acid release because of its antagonistic effect on histamine-2 receptors in pylori-ligated rats, but curcumin did not show this antagonistic effect^6^. Therefore, it was necessary to evaluate the interaction of the turmeric extract and its principal metabolite, curcumin, with ranitidine to determine whether the concomitant administration is favorable.

The interaction analysis, performed via the combination index and isobologram method^20^, shows that when the extract or the curcumin is administered before ranitidine (in 1:1 proportion), there is a subadditive gastroprotective effect (Fig. 5). Furthermore, the isobolographic analysis demonstrates that the *Dm Exp* of the combination with TAE or curcumin (31.11 and 31.79 mg/kg, respectively) was higher than the theoretical value (8.70 and 9.19 mg/kg), revealing a statistically significant difference (Fig. 4A,B).

When the rat stomachs were dissected, we observed the characteristic yellow color of both the extract and curcumin coating the mucosal layer. In order to evaluate a possible physicochemical interaction that would explain the antagonistic effect of the extract or the curcumin with ranitidine; the combinations were tested *in vitro* (0.1 M HCl, 37 °C, 2 h). The LC/MS analysis did not show any changes in the retention time or new signals that could suggest a chemical interaction (see supplemental information). As mentioned above, the barrier formed by TAE or curcumin could interfere with the gastroprotective effect of ranitidine. To assess this, we evaluated the mucoadhesion of the extract and the curcumin using a rheological method. The results shown in Fig. 6 suggest that both treatments present mucoadhesion in a simulated gastric medium (pH 1.6). The rheological parameters show synergism (positive ΔGʹ and ΔGʹʹ) in the TAE-mucin and curcumin-mucin mixtures. Some studies have reported that the “pseudo-solid” behavior of the mixtures is related to the interaction between the components of the mixture, where the viscous character dominates along the entire frequency range (Gʹʹ > Gʹ). However, in the TAE-mucin mixture, Gʹʹ was larger than Gʹ with a plateau independent to frequency^25^, which is possibly linked with mucoadhesion. The results of this study suggest that, in addition to the reported mechanism of action of the *C. longa* extract and curcumin, their mucoadhesion could be considered as a physical barrier mechanism related to wound and gastric ulcer healing. To corroborate whether the curcumin mucoadhesion was responsible for the antagonism, we decided to invert the sequence of administration. Ranitidine was administered 15 min before the curcumin to ensure its absorption. Figure 3G,J, corresponding to the TAE-ranitidine and curcumin-ranitidine combinations, present a larger damage area than Fig. 3H,K, which are the ranitidine-TAE and ranitidine-curcumin combined treatments (inverse administration). The *Dm Exp* of ranitidine-TAE and ranitidine-curcumin (7.49 and 7.66 mg/kg, respectively) was not statistically different from the *Dm Theo* (8.70 and 9.19 mg/kg) (Fig. 4C); demonstrating that the physical barrier formed by the TAE or curcumin, when interacting with mucin, is responsible for the antagonism on the ranitidine effect. These results are of great relevance for patients that consume turmeric as a complementary or alternative medicine for peptic ulcer or other illnesses, as it should be recommended that the administration of the antiulcer drug occur at least 15 min before turmeric to avoid an antagonistic effect.

Contrastingly, the co-administration of TAE or curcumin with bismuth subsalicylate (1:1 fixed ratio) did not show any pharmacologic interaction (Figs 7 and 8). The *Dm Exp* for the combinations was not statistically different than the *Dm Theo*. The *CI* analysis demonstrates a dual interaction effect for both combinations, antagonistic for *fa* < 0.5 and additive for *fa* > 0.5 (Fig. 7 and Table 1). This *CI* behavior indicates that doses higher than the *Dm* are adequate for the co-administration of the extract or curcumin with bismuth subsalicylate, and caution should be exerted with doses below the *Dm*. In pursuance of evaluating a possible chemical interaction between the curcumin and bismuth subsalicylate, we performed an *in vitro* assay where both compounds were mixed for 2 h at their corresponding *Dm* concentration in an acid environment (0.1 M HCl; 37 °C). Then the samples were analyzed by infrared spectroscopy. The spectra did not show any new functional groups or bonds between the compounds that would indicate a chemical interaction (see supplemental information). Therefore, it is necessary to further investigate the antagonistic mechanism observed at doses lower than the *Dm*. It has been suggested that prostaglandin induction and bicarbonate secretion are part of the molecular mechanism of bismuth salts; this could be involved in the *CI*-plot behavior^18^. On the other hand, the additive effect observed at the *Dm* could be related with the action mechanism of the treatments: the main mechanism of bismuth salts is coating the ulcer crater to improve wound healing, and the mucoadhesion of TAE or curcumin could also further improve the healing. In this case, both treatments share the same action mechanism; therefore, their gastroprotective behavior has an additive effect on treatments.

The ulcerative mechanism of the ethanol-induced injury model involves ROS^26^. The results in this work show that the extract and the curcumin are potent gastroprotective agents in this model that is likely explained by the antioxidant effect reported for curcuminoids^10^. The calculated *Dm* for curcumin, ranitidine and bismuth subsalicylate (Table 1) indicates that the *C. longa* extract is the most potent gastroprotective agent among the evaluated treatments.

## Conclusion

The *CI-*isobologram method demonstrates that when the extract or curcumin were administered before ranitidine (1:1), there is a subadditive interaction. However, when the administration sequence was inverted, the extract or curcumin did not affect the ranitidine gastroprotective action. The rheological results suggest that the mucoadhesion of the extract and curcumin is involved in their antagonistic action mechanism. Therefore, the concomitant use of turmeric or curcumin with other drugs should be carefully evaluated; we would recommend ingesting the turmeric or curcumin at least 15 min after the other drugs. Our results suggest that the mucoadhesion could be the mechanism for the wound and gastric ulcer healing, in addition to those reported for turmeric and curcumin. Additionally, the combinations with bismuth subsalicylate showed an additive effect in both cases, indicating there is no pharmacologic interaction in the ethanol-induced ulceration model.

Moreover, we demonstrated that the *Curcuma longa* extract and curcumin are an effective treatment to prevent ethanol-induced stomach damage. Both compounds are more potent gastroprotective agents than ranitidine and bismuth subsalicylate.

## Methods

### Plant material

Dry ground powder of *Curcuma longa* L. root was donated by Laboratorios MIXIM, S. A. de C. V. (Brach number 11590612), from which curcumin was isolated for biological testing.

### Curcumin isolation and identification

The *Curcuma longa* L. acetone extract (TAE) was obtained by Soxhlet reflux of the dry and ground plant material (1.5 kg) with acetone (3 L) for 6 hours, yielding 270 g of TAE. For curcumin isolation, the extract (100 g) was separated by column chromatography with 1 kg of silica gel 60 (0.063–0.200 mm, MERCK^®^). The column (90 × 7 i.d. cm) was eluted with hexane, followed by hexane: chloroform (1:1 to 1:9), then chloroform, and chloroform: methanol (99.5:0.5 to 97:3). Curcumin (Fig. 1) was isolated from the fractions eluted with chloroform and chloroform: methanol, as indicated by thin layer chromatography. The purification and crystallization of curcumin were carried out as follows. The crude curcumin was dissolved in hot methanol (60 °C), then ten times volume of cold hexane (−20 °C) was added and allowed to cool (4 °C) for 24 h. The crystals were filtered and washed with cold hexane, yielding 3.9 g of curcumin. The melting point of the obtained compound, measured with a Fisher-Johns apparatus, agrees with the reported value^27^. Liquid chromatography-mass spectrometry (LC/MS) analysis indicates a 99% purity. The mass spectrum of the curcumin was obtained by direct injection using a positive electronic impact (EI^+^) ionization source. The molecular ion showed a m/z 368 (M + 1)^+^; the most abundant fragment ion was m/z 177 (M + 1)^+^, in concordance with the literature^28^. The ^1^H and ^13^C NMR spectra were recorded in deuterated methanol (CD_3_OD) using a Varian Unity Plus 400 spectrometer at 400 MHz (^1^H) and 125 MHz (^13^C), using tetramethylsilane (TMS) as the internal standard.

^1^H-NMR (CD_3_OD) δ: 7.6 (H-1/7, d, *J* = 15.8 Hz); 7.24 (H-9/15, s); 7.14 (H-13/19, d, *J* = 8.3 Hz); 6.85 (H-12/18, d, *J* = 8.2 Hz); 6.66 (H-2/6, d, *J* = 15.8 Hz); 5.99 (H-4 *keto-enol form*, s); 4.59 (H-4 *diketo form*, s); 3.94 (OMe-10/6, s). ^13^C-NMR (CD_3_OD) δ: 182.53 (C-3); 182.58 (C-5); 148.24 (C-11/17); 147.18 (C-10/16); 139.88 (C-1/7); 126.35 (C-8/14); 121.86 (C-13/19); 120.02 (C-2/6); 114.33 (C-12/18); 109.51 (C-9/15); 99.73 (C-4); 54.22 (OMe-10/16).

The LC/MS analysis of TAE and curcumin as well as the mass and NMR spectra of curcumin are shown in the supplemental information.

### Drugs

Bismuth (III) subsalicylate (batch number MKBV4704V) and type III mucin from porcine stomach were purchased from Sigma Aldrich (St Louis, MO, USA). Ranitidine was donated by HELM de México S. A. (batch number RH4570316, 99.6% purity).

### Preparation of the suspensions for curcumin and extract administration

Individual suspensions of the pure compound and the extract were prepared as follows to facilitate oral administration. One hundred milligrams of curcumin or TAE were combined with 0.25 mL of tween 80, 0.25 mL of Span 20 and 1 mL of distilled water, and then stirred for 30 min at 338.2 × ***g*** with a homogenizer (ULTRATURAX^®^). Afterwards, distilled water was added at 0.5 mL/min to obtain a final volume of 10 mL, and constant stirring was maintained for additional 90 min. The suspensions showed viscoelastic characteristics and a non-Newtonian behavior. The particle size was measured (570 to 740 nm) by a Zetasizer ZEN ZS 3600 (Malvern Panalytical Co., UK). The suspensions were stored at room temperature away from sunlight.

### Rheological mucoadhesion evaluation

Mucoadhesion is the attachment of synthetic or biological macromolecules to a mucus layer by non-covalent molecular interactions^29^. The mucoadhesion of curcumin and the extract was evaluated using the rheological method previously reported by Hägerström *et al*.^30^. This methodology is based on the difference between the elastic (Gʹ) and viscous (Gʹʹ) moduli of the suspensions under oscillatory flow in a linear viscoelastic regime at 37 °C. The synergism parameters were estimated from Eqs. 1 and 2 (ΔGʹ y ΔGʹʹ); where Gʹ*sus* and Gʹʹ*sus*; Gʹ*muc* and Gʹʹ*muc*; and Gʹ*mix* (Gʹʹ*mix*) respectively represent the elastic and viscous moduli of the suspensions, mucin, and the mixture of mucin with curcumin or the extract. According to Hägerström et al. (2000), positive values of ΔGʹ and ΔGʹʹ indicate there is an interaction between the components in the sample, thus mucoadhesion occurs^30^.

1 $$\Delta G^{\prime} ={G}_{mix}^{^{\prime} }-({G}_{sus}^{^{\prime} }+{G}_{muc}^{^{\prime} })$$

2 $$\Delta G^{\prime\prime} ={G^{\prime\prime} }_{mix}-({G}_{sus}^{^{\prime\prime} }+{G}_{muc}^{^{\prime\prime} })$$

The mucin was dispersed under magnetic stirring for 14 h at room temperature in a simulated gastric medium (HCl aqueous solution, pH 1.6). The dispersion was then mixed with either curcumin or the extract suspension for a final mucin concentration of 5% (w/w). The suspensions were diluted at 3% (w/w) in the sample mixtures for the rheological evaluation. The mixtures were maintained under stirring for 30 min before testing. The rheological measurements were carried out using a controlled stress TA Instruments Discovery HR3^®^ rheometer with concentric cylinder geometry (21.96 mm external diameter, 20.38 mm inner diameter, 59.90 mm height and 500 μm of space between cylinders). The individual components and mixtures were characterized in the oscillatory flow mode from 1 to 100 (rad s^−1^) in the viscoelastic linear region (3% strain) at 37 ± 1 °C (circulating water bath, Cole-Parmer Polystat, and Peltier AR-G2).

### Animals

Male Wistar rats (250–300 g; 8 weeks old, n = 258) purchased from Envigo (Envigo RMS, S.A. de C.V., Mexico) were maintained at constant temperature (22 ± 2 °C) with free access to water and food. The rats were isolated and fasted in a metal cage with wire-net floor to avoid ingestion of feces and sawdust for 14 hours before the experiments; they had free access to water.

### Compliance with ethical standards

All applicable international, national and/or institutional guidelines for the care and use of animals were followed. Animal care and procedures were conducted in conformity with the Mexican Official Norm for Animal Care and Handing (NOM-062-ZOO-1999). This study was conducted under the supervision of the local Ethics Committee for the Use of Animals in Pharmacological and Toxicological Testing (CICUAL/147/16, 2016).

### Ethanol-induced gastric ulcer in rats

The ethanol-induced ulcer model has been previously reported^31^. The individual treatments (ranitidine, bismuth subsalicylate, curcumin or TAE) or combined (curcumin-ranitidine, curcumin-bismuth subsalicylate, TAE-ranitidine, TAE-bismuth subsalicylate) were orally administered 30 min before the alcohol-induced gastric damage (absolute ethanol, 1 mL). It is important to notice that for the combined treatments, each compound was individually administered. First, the TAE or curcumin were given to the rats 5 min before the antiulcer drug. Then, the sequence was inverted, and the antiulcer drugs were administered 15 min before the TAE or curcumin. The control group was treated with the vehicle only. Two hours after ethanol administration, the rats were sacrificed in a CO_2_ chamber, and their stomachs dissected and fixed with 10 mL of paraformaldehyde 4% for 5 min. Then, the stomachs were opened along the greater curvature and washed with a saline isotonic solution. A picture was taken using a digital microscope (Celestron 44302-A), to determine the damaged area (mm^2^), using the software Image J (Rasband, W.S., Image J, U.S., National Institutes of Health, Bethesda, Maryland, USA, http://imagej.nih.gov/ij/, 1997–2004).

### Gastroprotective effect and median-effect dose (*Dm*) determination for single treatments

The orally-administered individual drugs were tested at different doses to build a dose-dependent effect curve. Curcumin and TAE were evaluated at 10^−3^, 10^−2^, 10^−1^, 1 and 10 mg/kg; ranitidine at 1, 3, 10, 30, 100 and 300 mg/kg; and bismuth subsalicylate at 1, 10, 30, 100 and 177 mg/kg.

The first step for the interaction analysis was to calculate the median-effect dose (*Dm*) according to the *Median-Effect Equation* (Eq. 3) described by Chou and Talalay in 1976, for single drugs^20^. The *Dm* is defined as the dose when the affected fraction of the system (*fa*) is 0.5 (the dose that shows 50% of gastroprotection against the ethanol-induced gastric damage). In Eq. 3, *D* corresponds to each drug dose, *fu* is the unaffected fraction (1-*fa*), and *m* denotes the graph shape (*m* = 1 is for hyperbolic, *m* > 1 for sigmoidal and *m* < 1 for flat-sigmoidal dose-effect curves).

3 $$\frac{fa}{fu}={(\frac{D}{Dm})}^{m}$$

When plotting *x* = *log(D*) *vs y* = *log [fa/(1-fa)]* (Eq. 4), it linearizes the dose-effect curves to easily calculate the *Dm* and the *m* is calculated as the linear coefficient of the graph cited.

4 $$Log[\frac{fa}{1-fa}]=m\,Log(D)-mLog(Dm)$$

Once the *Dm* was calculated for each drug, the combined treatments were evaluated according to the experimental design shown in Table 2. The combinations tested were TAE-ranitidine, TAE-bismuth subsalicylate, curcumin-ranitidine, and curcumin-bismuth subsalicylate at 1:1 considering the calculated *Dm* for each drug^20^.

### Isobolographic analysis

The isobolographic analysis is a graphic representation of the doses of different drugs that have a specific effect, e.g., the median effect (*Dm*). A normalized isobologram is constructed from the (*D*)_1_/(*Dm*)_1_ of drug 1 *vs* (*D*)_2_/(*Dm*)_2_ of drug 2. Equation 5 presents the definition of additivity.

5 $$\frac{{(D)}_{1}}{{(Dm)}_{1}}+\frac{{(D)}_{2}}{{(Dm)}_{2}}=1$$

According to this equation, the maximum value for each drug is 1 in the *x-* and *y-*axis; a line called isobole or “additive line” connects these points and represents all the proportions in the combination that shows a theoretical additive effect. If the combination indicates there is an additive effect, the data points fall on this line. If the combination data points fall on the lower left or the upper right section, there is a superadditive (synergism) or subadditive (antagonism) interaction, respectively. The experimental points represent the median doses (*Dm*) of the drug combination.

Equations 6 and 7 correspond to concave and convex isobole, respectively. The area between the concave and convex lines defines the isobologram additive area. In Eqs 6 and 7 “*a*” values are the coordinates in the abscise (between 0 and 1 from the (*D*)_1_/(*Dm*)_1_ ratio), and “*b*” values are the coordinates in ordinate axis (between 0 and 1 from the (*D*)_2_/(*Dm*)_2_ ratio).

6 $$b=D{m}_{2}{(\frac{{D}_{1}-a}{D{m}_{1}})}^{{m}_{1}/{m}_{2}}$$

and

7 $$b={D}_{2}-\frac{D{m}_{2}}{{(\frac{D{m}_{1}}{a})}^{{m}_{1}/{m}_{2}}}$$

### Combination-index determination

The combination index (*CI*) for two drugs was determined according to Eq. 8 ^20^:

8 $$CI=\frac{{(D)}_{1}}{{(Dx)}_{1}}+\frac{{(D)}_{2}}{{(Dx)}_{2}}=\frac{{(D)}_{1}}{{(Dm)}_{1}{[\frac{fa}{1-fa}]}^{1/m}}+\frac{{(D)}_{2}}{{(Dm)}_{2}{[\frac{fa}{1-fa}]}^{1/m}}$$

A *CI* = 1 indicates there is an additive interaction; the superadditive and subadditive interactions correspond to *CI* < 1 and *CI* > 1. In Eq. 8, *Dx* represents the individual drug dose for reaching a specific percentage of effectiveness, and *D* is the dose of each drug in the combination that produces this effectiveness.

One of the advantages of the *CI*-plot, compared to the isobologram, is that it allows to analyze all *fa* levels simultaneously with any number of drugs in the combination.

### Statistical analysis

Statistically significant differences (*p* < 0.05) were interpreted as a synergism interaction if (*D*)_1_/(*Dm*)_1_ + (*D*)_2_/(*Dm*)_2_ was significative <1 and as subadditive interaction if (*D*)_1_/(*Dm*)_1_ + (*D*)_2_/(*Dm*)_2_ was significative >1. No statistical difference corresponded to an additive effect^23^.

The theoretical and experimental *Dm* of the combinations were compared based on the Mann-Whitney test to establish statistical difference (*p* < 0.05). The statistical difference between the treatments and the damaged control was determined by a One-way ANOVA and a *post hoc* Dunnett’s test.

## Supplementary information

Supplementary information
