# Supplementary material for: Mucoadhesive effect of Curcuma longa extract and curcumin decreases the ranitidine effect, but not bismuth subsalicylate on ethanol-induced ulcer model
Source: Sci Rep. 2019 Nov 12;9:16622. doi: 10.1038/s41598-019-53089-2 (PMC6851106; doi:10.1038/s41598-019-53089-2)
Supplement: Supplementary file 1 — Supplementary information [file 41598_2019_53089_MOESM1_ESM.docx]

**Supplemental information.**

**Mucoadhesive effect of *Curcuma longa* extract and curcumin decreases the ranitidine effect, but not bismuth subsalicylate on ethanol-induced ulcer model.**

Orona-Ortiz Alejandra^a^, Medina-Torres Luis ^a^, Velázquez-Moyado Josué A.^a^, Pineda-Peña Elizabeth A.^a^, Balderas-López José Luis^a^, Bernad-Bernad María Josefa^a^, Tavares Carvalho José Carlos^b^, Navarrete Andrés^a*^

*^a^Facultad de Química, Departamento de Farmacia. Universidad Nacional Autónoma de México. Ciudad Universitaria Coyoacán 04510, Ciudad de México, México .*

*^b^Laboratorio de Pesquisa em Farmacos, Curso de Farmacia, Departamento de Ciências Biológicas e da Saúde, Universidade Federal do Amapá, Macapá, AP, Brazil.*

***Corresponding author**:

Dr. Andrés Navarrete Castro

Facultad de Química, Departamento de Farmacia.

Universidad Nacional Autónoma de México

Ciudad Universitaria, Coyoacán 04510. México D.F.

México

Tel.: +55 56 22 52 91

Fax: +55 56 22 53 29

e-mail: anavarrt@unam.mx


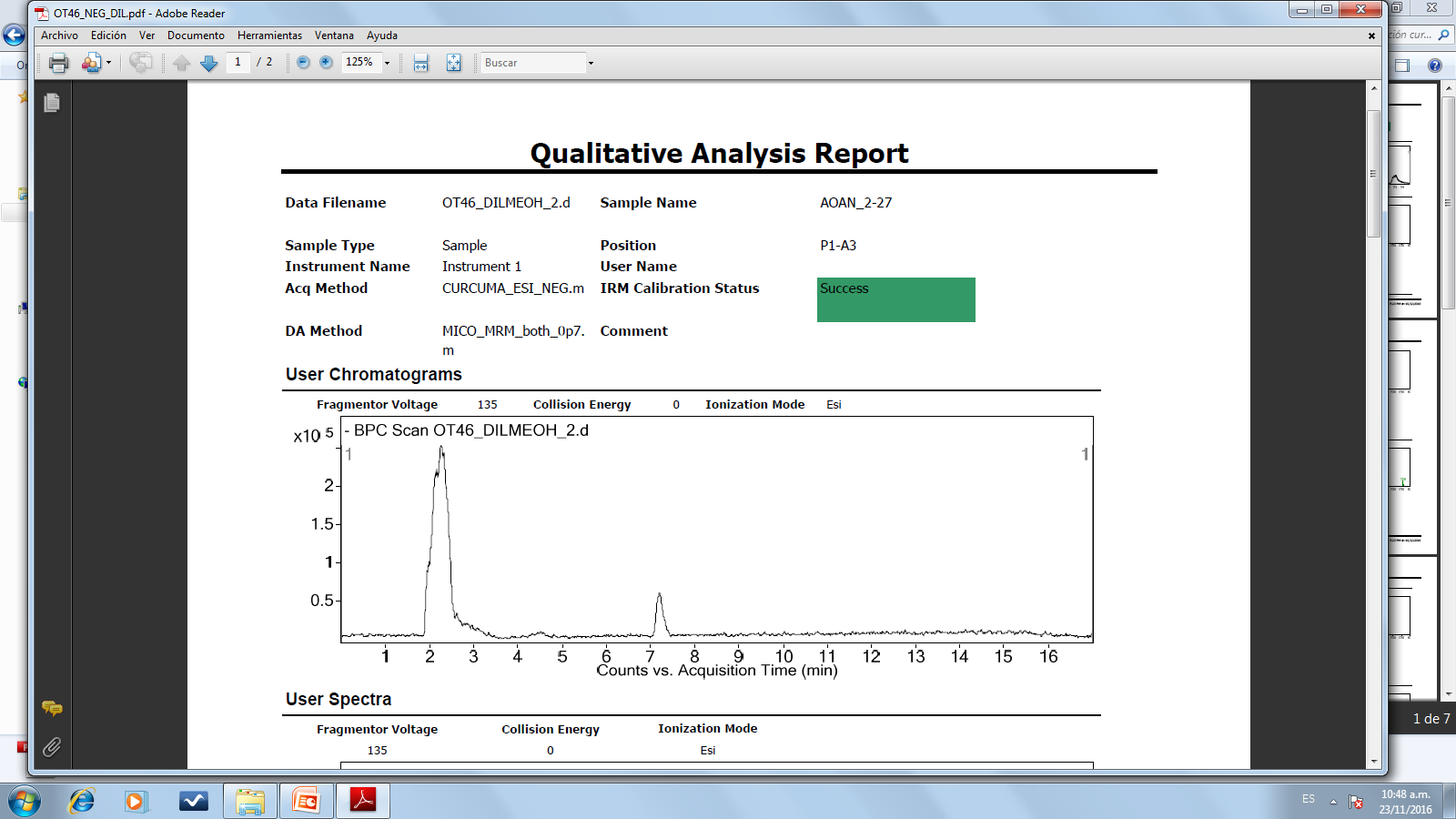


**A**


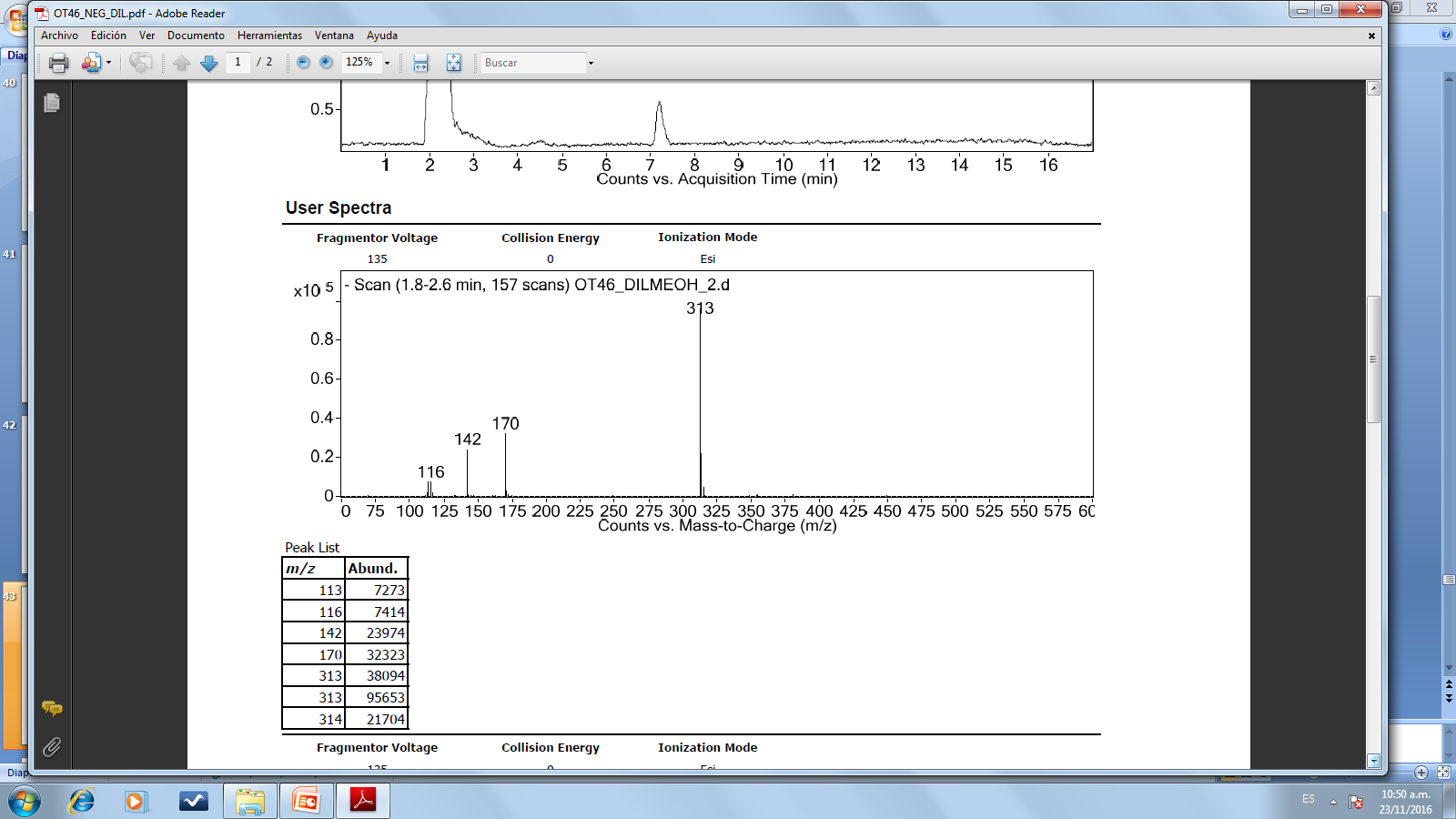


**B**

**C**

**Figure S1.** Curcumin-ranitidine mixture analyzed by LC/MS to establish a chemical interaction, using a 1200 Series Agilent^®^ HPLC hyphenated to a Triple Quad LC/MS 6410 Agilent^®^ Mass Spectrometer with ESI source. The column employed was a ZORBAX Extend-C18 (150 × 4.6 mm i.d..; 5 μm, Agilent^®^), elution conditions were: temperature 25ºC, flow rate 0.5 mL/min, system elution was achieved by gradient stating whit formic acid in water (0.1%):acetonitrile (50:50) to gradually change formic acid in water (0.1%):acetonitrile (5:95), elution time 22 min. Before injection, 5 mg of extract was diluted in methanol (0.9 mL) with formic acid (0.1 mL), then it was sonicated by 10 min and centrifuged by 10 min more at 22000×***g***. 200 μL of the prepared sample was diluted with 450 μL of formic acid in water (0.1%):acetonitrile (80:20) for injection. Curcuminoids detection was using a negative ESI at 350 ºC source temperature, with a gas flow rate of 8 L/min, capillarity voltage was 4 kV for the negative mode. The three principal curcuminoids were identified by their molecular ions (M-H)^-^ and fragmentation patterns.(**A**) Chromatographic profile of curcumin-ranitidine mixture (0.1M HCl). (**B**) Mass spectrum of the signal at 1.8-2.6 min of retention time, identified as ranitidine with a molecular ion of m/z 313 (M-1)^-^. (**C**) Mass spectrum of the signal at 7.0-75. min of retention time, identified as curcumin with a molecular ion of m/z 367 (M-1)^-^.

**Figure S2.** The blue continue line represents the infrared spectrum of curcumin-bismuth subsalicylate mixture, the red continue line represents the spectrum of curcumin alone and the black continue line is the spectrum of bismuth subsalicylate alone (all samples were placed in 0.1M HCl for 2 h at 37º C, to simulate gastric conditions).

Dry ground powder of *Curcuma longa* L. (Zingiberaceae) root was donated by Laboratorios MIXIM, S. A. de C. V. (Brach number **11590612**), from which curcumin was isolated for biological testing and turmeric acetone extract was obtained.

**A**

**B**

**C**

**D**

**Figure S3.**Turmeric acetone extract was analyzed by LC/MS to establish the relative composition of the major curcuminoids, using a 1200 Series Agilent^®^ HPLC hyphenated to a Triple Quad LC/MS 6410 Agilent^®^ Mass Spectrometer with ESI source. The column employed was a ZORBAX Extend-C18 (150 × 4.6 mm i.d..; 5 μm, Agilent^®^), elution conditions were: temperature 25ºC, flow rate 0.5 mL/min, system elution was achieved by gradient stating whit formic acid in water (0.1%):acetonitrile (50:50) to gradually change formic acid in water (0.1%):acetonitrile (5:95), elution time 22 min. Before injection, 5 mg of extract was diluted in methanol (0.9 mL) with formic acid (0.1 mL), then it was sonicated by 10 min and centrifuged by 10 min more at 22000×***g***. 200 μL of the prepared sample was diluted with 450 μL of formic acid in water (0.1%):acetonitrile (80:20) for injection. Curcuminoids detection was using a negative ESI at 350 ºC source temperature, with a gas flow rate of 8 L/min, capillarity voltage was 4 kV for the negative mode. The three principal curcuminoids were identified by their molecular ions (M-H)^-^ and fragmentation patterns.(**A**) Chromatographic profile of turmeric acetone extract. (**B**) Mass spectrum of the signal at 7.0-7.3 min of retention time, identified as bisdemethoxicurcumin with a molecular ion of m/z 307 (M-1)^-^. (**C**) Mass spectrum of the signal at 7.4-7.7 min of retention time, identified as demethoxicurcumin with a molecular ion of m/z 337 (M-1)^-^. (**D**) Mass spectrum of the signal at 7.7-8.1 min of retention time, identified as curcumin with a molecular ion of m/z 367 (M-1)^-^.

**A**

**B**

**C**

**Figure S4.**LC/MS analysis of curcumin by negative ESIionization mode. (**A**) Chromatographic profile of curcumin. (**B**) Mass spectrum of the signal at 4.2-4.6 min of retention time, identified as curcumin (*diketo* form) with a molecular ion of m/z 367 (M-1)^-^. (**C**) Mass spectrum of the signal at 7.6-8.1 min of retention time, identified as curcumin (*keto-enol* form) with a molecular ion of m/z 367 (M-1)^-^.

**Figure S5.** Curcumin: ^1^H-NMR (CD_3_OD)δ: 7.6 (H-1/7, d, *J*=15.8 Hz); 7.24 (H-9/15, s); 7.14 (H-13/19, d, *J*=8.3 Hz); 6.85 (H-12/18, d, *J*=8.2 Hz); 6.66 (H-2/6, d, *J*=15.8 Hz); 5.99 (H-4 *keto-enol form,* s); 4.59 (H-4 *diketo form,* s); 3.94 (OMe-10/6, s), ^13^C-NMR (CD_3_OD)δ: 182.53 (C-3); 182.58 (C-5); 148.24 (C-11/17); 147.18 (C-10/16); 139.88 (C-1/7); 126.35 (C-8/14); 121.86 (C-13/19); 120.02 (C-2/6); 114.33 (C-12/18); 109.51 (C-9/15); 99.73 (C-4); 54.22 (OMe-10/16).


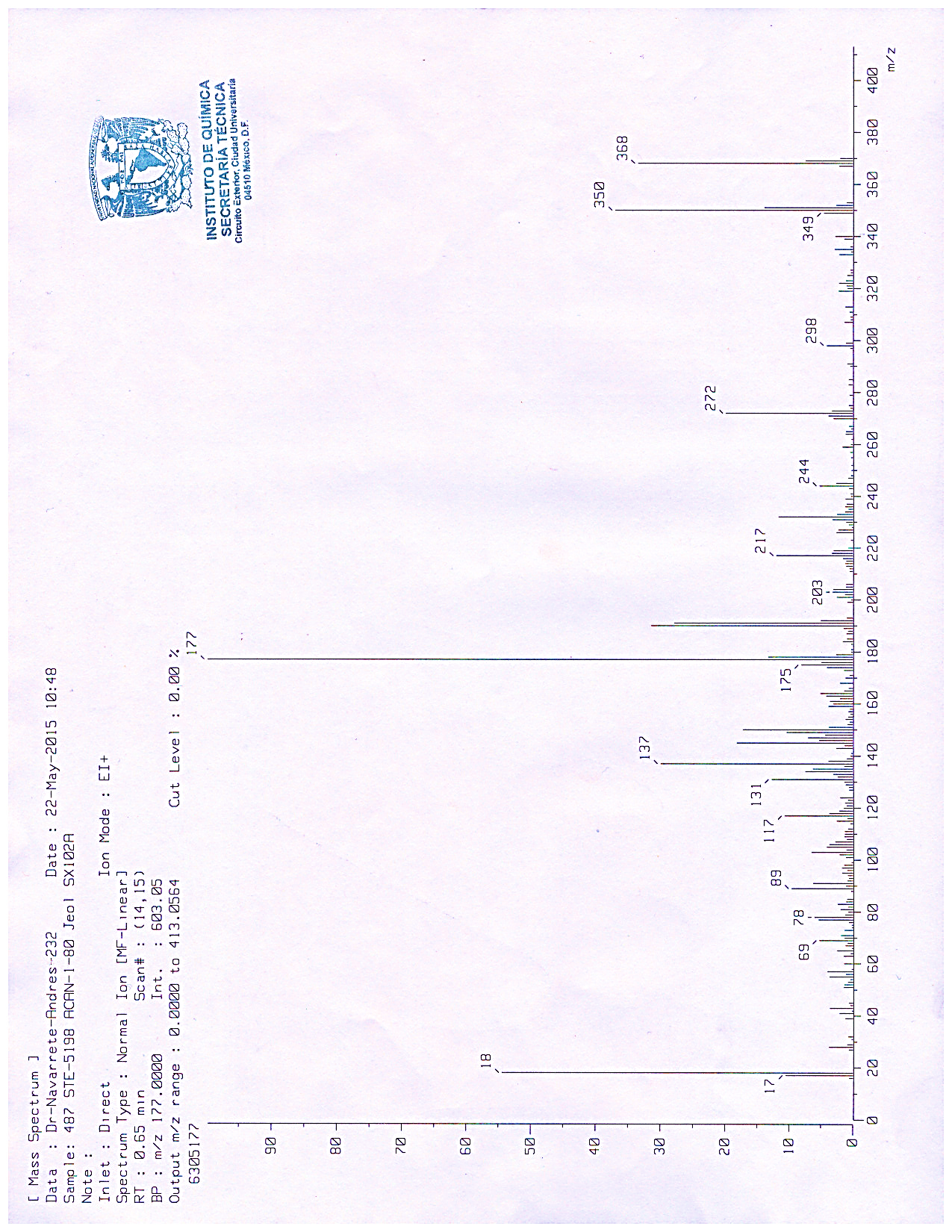


**Figure S6.** Mass spectra from curcumin. The ionization modewas positive electronic impact (EI^+^).
